# How people with brain injury run and evaluate a SLAM‐based smartphone augmented reality application to assess object‐location memory

**DOI:** 10.1002/pchj.784

**Published:** 2024-06-18

**Authors:** Magdalena Mendez‐Lopez, M.‐Carmen Juan, Teresa Burgos, Marta Mendez, Camino Fidalgo

**Affiliations:** ^1^ Departamento de Psicología y Sociología Universidad de Zaragoza, Facultad de Ciencias Sociales y Humanas Teruel Spain; ^2^ Instituto de Investigación Sanitaria Aragón (IIS Aragón) Zaragoza Spain; ^3^ Instituto Universitario de Automática e Informática Industrial Universitat Politècnica de València Valencia Spain; ^4^ Instituto de Neurociencias del Principado de Asturias (INEUROPA) Oviedo Spain; ^5^ Departamento de Psicología Universidad de Oviedo, Facultad de Psicología Oviedo Spain

**Keywords:** augmented reality, disability, neuropsychological practice, spatial memory, usability

## Abstract

Augmented reality (AR) technology allows virtual objects to be superimposed on the real‐world environment, offering significant potential for improving cognitive assessments and rehabilitation processes in the field of visuospatial learning. This study examines how patients with acquired brain injury (ABI) evaluate the functions and usability of a SLAM‐based smartphone AR app to assess object‐location skills. Ten ABI patients performed a task for the spatial recall of four objects using an AR app. The data collected from 10 healthy participants provided reference values for the best performance. Their perceptions of the AR app/technology and its usability were investigated. The results indicate lower effectiveness in solving the task in the patient group, as the time they needed to complete it was related to their level of impairment. The patients showed lower, yet positive, scores in factors related to app usability and acceptance (e.g., mental effort and satisfaction, respectively). There were more patients reported on entertainment as a positive aspect of the app. Patients' perceived enjoyment was related to concentration and calm, whereas usability was associated with perceived competence, expertise, and a lower level of physical effort. For patients, the sensory aspects of the objects were related to their presence, while for healthy participants, they were related to enjoyment and required effort. The results show that AR seems to be a promising tool to assess spatial orientation in the target patient population.

## INTRODUCTION

Advances in technology allow people to live longer and maintain quality of life. In recent years, several studies have used technologies to develop programs to assess, prevent, or reduce cognitive or motor impairment caused by acquired brain injury (ABI) (Kang et al., 2008; van der Kuil et al., 2018; Wenk et al., 2022). For instance, computerized neuropsychological tests for assessing cognitive function have been developed to replace traditional pen‐and‐paper tests, which are time‐consuming and expensive (Zhang & Feinstein, 2016). However, neuropsychological tests (both pen‐and‐paper and computerized) are not representative of everyday situations and, therefore, cannot assess the level of cognitive function required to perform daily activities (Pugnetti et al., 1998). Patients may also have difficulties performing complex multitasking activities, whereas results from assessments using pen‐and‐paper neuropsychological tests do not produce these difficulties (see Bonato, 2012). In fact, some authors have highlighted that the main goal of any rehabilitation program should be to improve patients' quality of independent living through everyday activities (Hampstead et al., 2014). Virtual reality (VR) is increasingly recognized for its potential to overcome this problem. This technology has improved its ecological validity by allowing patients to immerse themselves in a simulation that reflects real‐life situations (Bohil et al., 2011).

Rehabilitation therapies, either cognitive or physical, need human resources, are expensive and take a long time. Patients often find these therapies tedious, repetitive, boring and demanding, which makes it difficult for them to complete rehabilitation programs. VR has emerged as a promising tool that can reduce healthcare costs and enhance the motivation and engagement of patients in rehabilitation programs (Tieri et al., 2018). There is evidence to support the effectiveness of VR as a rehabilitation therapy (Kang et al., 2008; Luque‐Moreno et al., 2015; Osumi et al., 2017).

Augmented reality (AR) technology allows virtual objects to be superimposed on the real‐world environment. It has great potential in many fields; in fact, AR has already been incorporated into several disciplines, such as psychology (Juan et al., 2005) and education (Furió et al., 2013). In the field of behavioral health, AR has been used as a therapeutic tool for a range of conditions, including phobias, anxiety, and autism spectrum disorder (Bakır et al., 2023; Toma et al., 2024).

In visual AR, the sense of sight is enhanced, and virtual objects are overlaid on the real‐world environment. From the psychology perspective, both VR and AR offer interesting advantages during the assessment. It is very easy for the experimenter/instructor to control variables and store the participants' responses (Juan et al., 2014). However, AR has some benefits compared to VR: (1) users' interact with both real‐world and virtual items in real time, thus enhancing their sensory perception of reality (Hugues et al., 2011); (2) AR does not induce cybersickness, a frequent side effect of VR that sometimes leads to nausea, vertigo, and vomiting (Johnson, 2005), which is especially important in rehabilitations that need repeated use of therapy or training apps (Bohil et al., 2011). Although AR has great potential, only a few pilot studies have used it as a treatment for motor impairment in Parkinson's disease (Tunur et al., 2019), stroke (de Assis et al., 2016; Mousavi Hondori et al., 2016), and cognitive decline (Hervas et al., 2014). Therefore, further research is needed to build an evidence base demonstrating the utility and effectiveness of VR and AR for clinical care and rehabilitation. Moreover, our app is based on simultaneous localization and mapping (SLAM) and can be used anywhere without requiring additional elements to be added to the real scene for tracking.

### Assessment of spatial memory using virtual and augmented reality

Spatial memory is a type of memory that is involved in navigation and spatial abilities and everyday tasks, such as remembering the location of objects (Burgess et al., 2001). Therefore, patients with spatial memory impairment suffer devastating effects in their lives. Several studies have addressed spatial orientation deficits—in both near and navigational space—in patients with brain injury (Piccardi et al., 2011; Rose et al., 1999). Furthermore, misplacing objects is a frequent presenting complaint of people with neurological impairment (Hampstead et al., 2011), which hints at the importance of the functional integrity of spatial memory processes in patients' quality of life. The development of spatial tasks to be carried out in the real world is difficult, and frequently these tasks do not involve participants navigating in a similar way to how they do so in real life (Fernandez‐Baizan et al., 2019).

AR technology allows the experimenter to superimpose virtual objects on the real world to supplement reality (Azuma et al., 2001). Thus, when participants perform tasks in AR conceived for wide spaces, they navigate similarly to how they do in real life (Munoz‐Montoya, Juan, et al., 2019). Despite its potential, very few studies have specifically investigated spatial memory performance in spatial tasks using AR (Juan et al., 2014; Mendez‐Lopez et al., 2016; Munoz‐Montoya, Fidalgo, et al., 2019; Munoz‐Montoya, Juan, et al., 2019; Peleg‐Adler et al., 2018). In particular, AR based on image targets distributed in the real environment has been used to assess short‐term spatial memory in children (Juan et al., 2014; Mendez‐Lopez et al., 2016). More recently, a SLAM‐based AR app run on a smartphone (Lenovo Phab 2 Pro) was used to assess spatial orientation of adults in a two‐story building (Munoz‐Montoya, Juan, et al., 2019). In this study, the participant must look for virtual objects located in a real building and remember their locations in order to place them in their correct real‐world locations later. Another study utilized AR to assess spatial memory but using auditory stimuli instead of visual ones. In this study, participants were required to remember the location of auditory stimuli while physically walking around a real environment and subsequently recall the location of those stimuli (Ponce et al., 2024).

### Attitudes of brain‐injured patients toward technology

ABI patients' acceptance of AR and their attitude toward it are crucial to ensure they use and adopt it. However, ABI patients may have deficits in motor and/or executive functions such as reasoning, planning, mental flexibility, and attention (Barker‐Collo & Feigin, 2006; Cicerone et al., 2000; McDonald et al., 2002), which may interfere with the use of new technologies (Wilson et al., 1989). In fact, although many ABI patients show an interest in electronic devices (Hart et al., 2004), they frequently experience difficulties using technology such as computers or telephones (Lindén et al., 2010; Lovgren Engstrom et al., 2010).

### The current study

Most of the research aimed at assessing spatial deficits in ABI patients had been performed using paper‐and‐pencil psychometric tests or VR tasks (Astur et al., 2002; Pallavicini et al., 2015). Regarding object location, impairments in ABI patients have been previously assessed by Kessels et al. (2002). However, in this study, the task was performed on a computer and did not accurately reflect functions in daily life. Currently, there is limited literature that has examined the use of AR in patients with ABI for the study of spatial memory. Additionally, little is known about patients' acceptance and their attitude toward AR.

The purpose of this study was to investigate: (1) objective parameters to assess whether the AR app could detect differences in object‐location skills between ABI patients and controls; (2) differences in quantitative and qualitative perceptions of the AR app between ABI patients and control participants; (3) possible associations between participants' characteristics, performance, and subjective perceptions of the task; and (4) the differences between groups in their preferences regarding the app and suggestions for its application in alternative contexts.

We hypothesized that ABI patients would be able to use the AR app, but they would perform the AR spatial task worse than healthy people. We also hypothesized that ABI patients would report positive perceptions of the AR app, but their scores would be lower than those of the healthy participants. Due to the limited literature on the subject, formulating hypotheses regarding the third and fourth objectives is not feasible.

## MATERIALS AND METHODS

### Participants

Ten ABI patients took part in the study. They were recruited from a care center for dependent adults. Twenty‐one eligible participants were identified by a professional at the center. Ten eligible patients meeting the inclusion criteria agreed to participate and were included in the study. Eleven eligible patients refused to participate. The recruitment and screening were done by the third author. The inclusion criteria were being patients with ABI, able to handle objects, with no visual or hearing impairment, no communication difficulties, and able to deal with the study protocol. The following instruments were used: the Mini‐Mental State Examination (MMSE), which measures the current level of cognitive function (23–10 = mild to moderate dementia and ≥24 = normal function) (Lobo et al., 1979); the Barthel Index (BI), which measures the level of dependence in everyday activities (<20 total, 21–60 severe, 61–90 moderate, and 91–99 slight dependence) (Mahoney & Barthel, 1965); and the Tinetti Mobility Test (TMT), which indicates the risk of falls (≤19 high, 19–24 moderate, and ≥24 low risk) (Tinetti et al., 1986). Clinicians determined the diagnosis process for ABI patients through a combination of neuroimaging, physical examination, and neurological examination. They recorded the diagnoses in the patients' medical history. Their characteristics are summarized in Table 1.

**TABLE 1:** Patient sample characteristics.

ID	Age	Sex	ABI type	Years after lesion	Side of lesion	Other diagnoses	MMSE	BI	Mobility impairment, (TMT)	Education	Use of smartphones to play
A	43	M	TBI	>20	R	–	24	80	Hemiparesis, use of crutch (20)	Primary	Never
B	44	M	TBI	>10	R	–	30	100	– (28)	Primary	Once a month
C	51	M	IS	>7	L	–	29	90	Ataxia, use of walker (17)	Secondary	Once a month
D	51	M	IS	>4	L	Aphasia	27	95	Hemiparesis (25)	Primary	Once a week
E	55	M	TBI	>4	L	–	30	100	– (27)	Primary	Never
F	58	M	IS	>7	R	KS	21	95	– (28)	Primary	Never
G	59	F	IS	>4	ND	KS	27	70	Feet deformity, use of wheelchair (1)	Primary	Never
H	60	M	IS	>4	R	–	28	55	Hemiplegia, use of wheelchair (11)	Secondary	Almost daily
I	60	F	IS	>4	ND	Aphasia	12	95	Ataxia, use of walker (18)	Primary	Never
J	62	M	IS	>4	R	–	30	15	Hemiplegia, use of wheelchair (1)	Primary	Almost daily

*Note*: Patients' identities are coded using letters. The BI and TMT scores were obtained 5 months before recruitment. The MMSE measures the current level of cognitive function (23–10 = mild to moderate dementia and ≥24 = normal function; maximum score is 30). The score of patients F and I on the MMSE suggests a mild to moderate dementia. The symbol ‘–’ indicates no particular difficulty or diagnosis.

Abbreviations: ABI, acquired brain injury; BI, Barthel index; IS, ischemic stroke; KS, Korsakoff's syndrome; L, left; MMSE, Mini‐Mental State Examination; ND, not defined; R, right; TBI, traumatic brain injury; TMT, Tinetti Mobility Test.

There were 10 healthy participants (healthy group) that were recruited from the university via on‐campus advertisements. This group was selected to provide reference values for participants with full competence to perform the task with the AR app. Inclusion criteria in the healthy group were people with a minimum age of 43 years (cut‐off point based on the age of the ABI group), no history of brain injury or severe disease that required hospitalization, no visual, motor or hearing impairment and no ongoing treatment with medications that could potentially cause cognitive or motor impairment. The group of healthy participants consisted of four men and six women between the ages of 44 and 60 (male: *M* = 45.75, *SD* = 2.36; female: *M* = 51.33, *SD* = 5.43). Of these participants, two had primary education, five had secondary education, and three had higher education. Regarding the frequency of using smartphones or tablets to play games, four reported never using them, three reported using them once a week, two reported using them once a month, and one reported using them almost daily. The ABI and healthy groups were comparable based on their age (*t*‐test: *p* = .07), the proportion of women participating in each group (two‐tailed Fisher's exact test: *p* = .17), and their frequency of using smartphones or tablets to play games (two‐tailed Fisher's exact test: *p* = 1). None of the participants had any prior experience with AR. All participants signed an informed consent form. The Ethics Committee of the Universitat Politècnica de València approved the study (P18_27_09_17).

### Assessment of object‐location memory with the AR app

**FIGURE 1:**
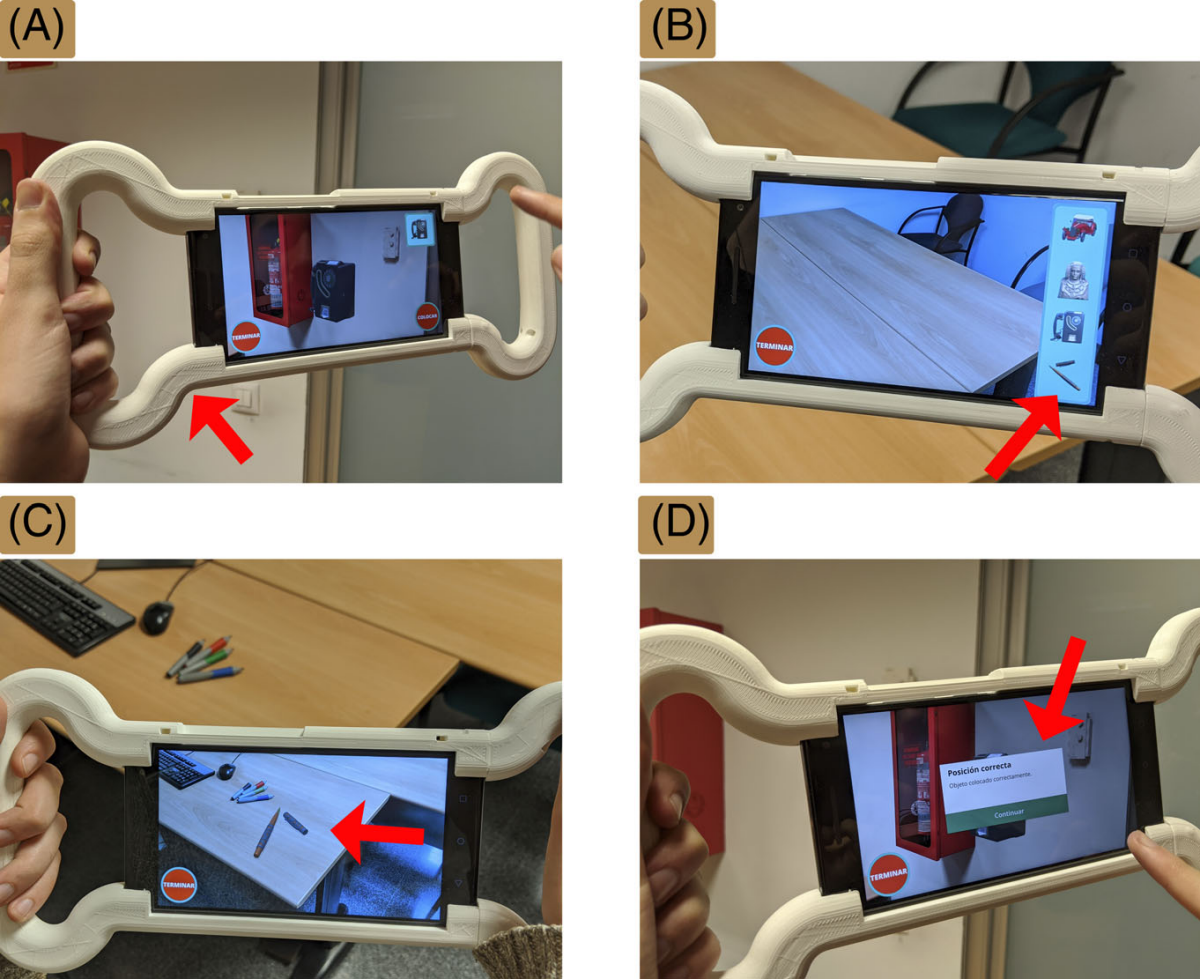
The AR app. (A) External case to facilitate handling. (B) The right side of the screen shows the four objects that had to be placed correctly. (C) Example of the adaptation of a virtual object to the surface of a table. (D) Message reporting success in placing an object.

The participants performed a short‐term memory task consisting of locating four virtual objects using an AR app (Munoz‐Montoya, Fidalgo, et al., 2019) (Figure 1). Before the spatial task, participants were given a short introductory training by one of the researchers. The introductory training lasted about 5 min and covered the basics of using the app, including instructions on how to locate a virtual object within a room and place it in a specific location. The short‐term memory task involved two phases. In the first phase, the participants learned the location of four virtual objects that were randomly distributed in a familiar real‐world leisure room of 40 m^2^. For this purpose, an examiner asked the participants to inspect the room, to look for four virtual objects and to remember their locations so they could place them in their correct real‐world location later (Figure 1A). The first phase ended when the participants had found the fourth object. In the second phase, the examiner asked the participants to place the objects in the correct location using the app. The four objects were shown on the right side of the screen (Figure 1B). The participants had to select the objects one by one and place them in the environment (Figure 1C). If the position was correct, the user was informed of their success (Figure 1D), the object was locked in that place, and disappeared from the list of available objects. If the position was incorrect, the participants were informed about the remaining available attempts (three per object). There was no specific time limit for completing the task.

There were five variables related to task performance: time in seconds to complete the task (Time), number of objects located correctly (Objects located), percentage of objects located correctly out of the four total objects (% Success), number of attempts made to place the four objects (Attempts made), and the Attempts made variable expressed as a percentage (% Attempts).

### Assessment of usability and acceptance of the AR app

The participants completed the usability and acceptance survey (UAS). The UAS consisted of 20 items measuring the following factors: enjoyment (Item 1); concentration (Item 2); usability (Items 3–5); competence (Item 6); calm (Item 7); expertise (Item 8); mental effort (Item 9); physical effort (Item 10); satisfaction (Items 11–12 and 19–20); sensory items (Items 13–14); and presence (Items 15–18). The following UAS factors were adapted from commonly used questionnaires: enjoyment, competence and calm (Intrinsic motivation inventory; McAuley et al., 1989); concentration, expertise and sensory aspects (Presence questionnaire; Witmer & Singer, 1998); usability (System Usability Scale; Brooke, 1996), and presence (Regenbrecht & Schubert, 2002; Slater et al., 1994). For mental effort, physical effort, and satisfaction, we included items based on previous research (Calle‐Bustos et al., 2017; Munoz‐Montoya, Juan, et al., 2019). The items were measured on a 7‐point Likert scale ranging from 1 ‘*Totally disagree*’ to 7 ‘*Totally agree*’. All were formulated positively except for items 9 and 10, for which a higher score indicated a lower level of mental and/or physical effort. We obtained the raw scores of each participant in each item of the survey and we calculated the mean score for each factor in each studied group.

We used Cronbach's alpha to assess the internal consistency of the UAS. The internal consistency of all the items in our sample was good, Cronbach's α = 0.95.

### Assessment of the positive aspects of the AR app and uses of AR technology

Additionally, the participants were asked two open‐ended questions to ascertain both the positive aspects of the AR app (question: ‘What did you like the most?’) and the perceived uses of this technology (question: ‘What do you think this technology could be used for?’). All participants' answers to the open‐ended questions were considered from qualitative and quantitative perspectives. Two of the co‐authors achieved this independently by reading the participants' answers to find possible categories or themes for subsequent classification. They then shared their results and jointly decided on the final categories to classify the answers into (Braun & Clarke, 2006). These categories were as follows: potential and characteristics of the technology/app, the objects, entertainment, therapy purposes, and other purposes. The two groups' frequency of preference for each category was compared.

### Procedure

All participants were called for an individual session of approximately 50 min. Only the ABI group was assessed for the MMSE (Lobo et al., 1979) at the beginning of the individual session. ABI and control groups completed the object‐location memory task using the AR app. Subsequently, they answered questions on their experience using smartphones to play games, the UAS, and the two open‐ended questions about the AR app and AR technology through a personal interview.

### Statistical analyses

Version 26.0 of IBM SPSS was used to perform the statistical analyses. We applied the Shapiro–Wilk test to check normality distribution of the dataset variables. The tests showed that only the variable measuring the time performing the AR task followed a normal distribution in each studied group. However, Levene's test showed that the variances between the groups were not homogeneous. Therefore, we decided to perform non‐parametric tests with the entire dataset, which are more suitable for distributions of this type.

Mann–Whitney tests were used to check differences between the two groups in the performance measures with the app and UAS factors. The parameter of effect size was *r*. Spearman's correlations were conducted separately for each group to inspect the relation between performance and the subjective parameters of the task with the AR app. In the ABI group, the levels of cognitive function, dependence and mobility were also considered as variables to calculate the correlations. Given the absence of variability in the healthy group, no correlations were calculated for the variables: % Success and % Attempts, and the UAS concentration and calm factors.

Fisher's exact test was used to compare the preference frequency of the ABI and control groups for each category derived from participants' responses to the open‐ended questions.

All *p*‐values were two‐tailed, and the level of significance was taken as *p* < .05.

## RESULTS

### Performance in the object‐location memory task using the AR app

Table 2 shows the time spent, objects located, and attempts made by each patient to complete the AR task.

**TABLE 2:** Performance data acquired by the app device in the object‐location memory task.

Patient	Time (s.)	Objects located (max. 4)	% Success	Attempts made (max. 12)	% Attempts
A	139.67	2	50	7	58.30
B	139.75	2	50	8	66.70
C	490.09	4	100	5	41.70
D	245.37	3	75	4	33.30
E	252.50	2	50	7	58.30
F	289.43	0	0	12	100
G	124.02	0	0	12	100
H	36.70	0	0	12	100
I	266.03	1	25	12	100
J	74.42	4	100	4	33.30
*M*	205.80	1.80	45.00	8.30	69.16
*SD*	*131.85*	*1.55*	*38.73*	3.43	*28.62*
Healthy participants					
*M*	106.48	4.00	100	4.00	33.30
*SD*	*25.59*	*0.00*	*0*	*0.00*	*0*
Sig.	*p = *.041		*p = *.001		*p < *.001

*Note*: Patients' identities are coded using letters. Time spent, objects located and attempts made to complete the AR task. Sig. = Significance of the comparison between groups (Mann–Whitney tests).

The mean of these measures obtained by the healthy group is also given as a reference. All the participants in the healthy group scored the highest level of competence in the variables related to the recall of the objects and accuracy in their location (i.e., success and attempts, respectively). The ABI group performed significantly slower than the healthy group (*U* = 23.00, *Z* = −2.04, *p* = .041, *r* = −0.45). The ABI group showed a worse recall of the location of the objects (% Success: *U* = 90.00, *Z* = 3.42, *p* = .001, *r* = 0.76), and was less efficient in performing the task than the healthy group, since the patients comprising this group needed more attempts to complete the AR task than the healthy participants in the healthy group (% Attempts: *U* = 5.00, *Z* = −3.74, *p* < .001, *r* = −0.84). The effect size was large for the differences found in the number of objects recalled and the accuracy in the location of these objects.

### Usability and acceptance of the app by the participants

Table 3 shows descriptive statistics of the UAS factors and the statistically significant differences between the ABI and healthy groups.

**TABLE 3:** Mean scores and standard deviations of the UAS factors and significance of the comparison between the ABI and healthy groups.

UAS factor (item #)	Groups:	ABI	Healthy	Sig.
	*n*	10	10	
**Enjoyment** (1. I really enjoyed doing this activity)	*M*	5.80	6.90	***p* = .005**
	*SD*	0.92	0.32	
**Concentration** (2. I have focused on the tasks that I had to do and not on the control mechanisms)	*M*	5.80	7	***p* < .001**
	*SD*	0.63	0	
**Usability** (3. It was easy to handle the app / 4. It was easy to learn how to use the app / 5. I do not need the help of an expert to use this app)	*M*	5.60	6.68	***p* = .001**
	*SD*	0.59	0.41	
**Competence** (6. I am happy with how I have done it)	*M*	5.40	6.90	***p* < .001**
	*SD*	0.52	0.32	
**Calm** (7. I have been calm during the experience)	*M*	5.90	7	***p* = .002**
	*SD*	0.87	0	
**Expertise** (8. At the end of the experience, I felt expert in handling the app)	*M*	5.70	6.20	*p* = .102
	*SD*	0.67	0.63	
**Mental Effort** (9. The use of the app did not require a great mental effort)	*M*	5.60	6.70	***p* = .002**
	*SD*	0.52	0.67	
**Physical Effort** (10. The use of the application did not require a great effort of arms and hands)	*M*	5.70	6.70	***p* = .005**
	*SD*	0.67	0.67	
**Sensory** (13. I was able to examine virtual objects closely / 14. I was able to examine virtual objects from different parts)	*M*	4.70	6.90	***p* < .001**
	*SD*	2.07	0.32	
**Presence** (15. There were moments during the experience when I thought the virtual objects were real/ 16. When you reflect and think about the experience, you remember virtual objects as objects that were in the room / 17. I had the feeling that the virtual objects were in the room / 18. I did not pay attention to the difference between virtual objects and the real world)	*M*	4.66	6.40	***p* = .004**
	*SD*	1.98	0.63	
**Satisfaction** (11. I would like to use the app again / 12. I would like to use this technology for other uses / 19. I liked how the virtual objects looked / 20. Rate the experience)	*M*	5.68	6.79	***p* = .002**
	*SD*	0.70	0.35	

*Note*: UAS: All the questions used a 7‐point Likert scale ranging from 1 ‘*Totally disagree*’ to 7 ‘*Totally agree*’ and were formulated in a positive manner, except for items 9 and 10. Sig. = Significance. Bold values indicate *p*‐values below the significance threshold (<.05; Mann–Whitney tests).

The patients considered the AR app as significantly less positive than the healthy participants in terms of usability (*U* = 94.00, *Z* = 3.38, *p* = .001, *r* = 0.75), enjoyment (*U* = 82.50, *Z* = 2.80, *p* = .005, *r* = 0.63), concentration (*U* = 95.00, *Z* = 3.79, *p* < .001, *r* = 0.85), competence (*U* = 98.00, *Z* = 3.89, *p* < .001, *r* = 0.87), calm (*U* = 85.00, *Z* = 3.13, *p* = .002, *r* = 0.70), mental effort (*U* = 89.00, *Z* = 3.14, *p* = .002, *r* = 0.70), and physical effort (*U* = 84.50, *Z* = 2.80, *p* = .005, *r* = 0.63). Regarding the assessment of the sensory and presence aspects of the virtual objects, the ABI group also reported significantly lower scores than the healthy group (*U* = 93.00, *Z* = 3.49, *p* < .001, *r* = 0.78 and *U* = 87.50, *Z* = 2.85, *p* = .004, *r* = 0.64, respectively). The ABI group was also less satisfied with the experience and the appearance of the virtual objects than the healthy group (satisfaction: *U* = 91.00, *Z* = 3.17, *p* = .002, *r* = 0.71). The only factor in which the groups did not differ was expertise (*U* = 69.50, *Z* = 1.63, *p* = .102, *r* = 0.36), since all the participants felt the same level of expertise handling the AR app at the end of the experience.

With regard to the Spearman's correlations, for reasons of brevity, only the most relevant correlation coefficients that reached significance are described. Spearman's correlations conducted in the group of healthy participants showed that the lower levels of mental and physical effort were significantly and directly related to sensory aspects (i.e., a good examination of the virtual objects, nearby and from different perspectives) and enjoyment of using the app (all, *r* = 0.74, *p* = .013). There were also significant direct relationships between enjoyment of using the AR app and sensory aspects (*r* = 1, *p* < .01). In the patients, the level of dependence (BI variable) was significantly related to the time patients spent performing the task (*r* = 0.63, *p* = .049). Enjoyment was significantly and directly related to the concentration (*r* = 0.69, *p* = .026) and calm (*r* = 0.76, *p* = .011) reported during the use of the app. Concentration and calm during the experience were also significantly related (*r* = 0.77, *p* = .088). Concentration was also significantly related to a higher level of satisfaction and a lower level of mental effort (*r* = 0.76, *p* = .011 and *r* = 0.77, *p* = .009, respectively). Usability was significantly related both to a higher level of competence and expertise (*r* = 0.80, *p* = .005 and *r* = 0.76, *p* = .010, respectively) and a lower level of physical effort (*r* = 0.76, *p* = .010). Finally, the presence of the virtual objects was significantly related to their sensory aspects (*r* = 0.98, *p* < .0001). Considering the correlation coefficients, the effect size for each correlation was large (Cohen, 1998).

Table 4 shows the participants' answers to the two open‐ended questions about what they liked most about the app and ideas they might offer for using the app in other contexts, which were grouped into categories. The frequency of preference between the two groups for each category was compared. No significant differences were observed between the patients and the healthy group in the categories: potential and characteristics of the technology/app (*p* = 1; odds ratio [OR]: 0.64), and the objects (*p* = .35; OR: 0.25) except for a marginal difference in the entertainment and therapy purposes categories, more preferred by the patients (both *p* = .07; OR: 9.33). Finally, the healthy group mentioned a greater number of additional alternative uses for this technology (other purposes: *p* = .02; OR: 0.05).

**TABLE 4:** Participants' answers and categories.

In reply to question 1: What did you like the most?	
**Category**	**(code) Answer**
Potential and characteristics of the technology/APP	**(C)** Using a smartphone for memory exercises is different
	**(H)** It is a quick game, but difficult …
	**(I)** It is a novelty, I had never used it
	**(2)** … the easy handling of the application
	**(3)** The possibility of imagining any object where I want it
	**(5)** The possibility of creating other different worlds, of expanding reality and living the whole world of possibilities that could be experienced
	**(7)** The ease with which a home could be decorated without the need for experts
The objects	**(A)** The objects were very well made
	**(J)** The objects were well made …
	**(1)** Being able to see virtual objects
	**(2)** The quality of the virtual objects …
	**(6)** Reality of the objects
	**(8)** That objects appear and disappear
	**(9)** Seeing the objects as if they were real
Entertainment	**(B)** It is short and fun, you need to focus a lot …
	**(D)** It is like a game, you have fun doing the exercises
	**(E)** It was entertaining …
	**(F)** That it is like a game
	**(G)** It was fun
	**(H)** It is a quick game …
	**(J)** … it was a short game
	**(4)** Is very enjoyable
	**(10)** I had fun placing the objects
In reply to question 2: What do you think this technology could be used for?	
**Category**	**(code) Answer**
Therapy purposes	**(A)** for workshops in centers
	**(B)** to recover from injuries
	**(C)** we would improve our memory
	**(E)** to practice with it
	**(F)** we can learn like this, it is more fun
	**(H)** for workshops
	**(I)** to learn how to orient yourself
	**(J)** to be used in workshops (app)
	**(1)** mental disorder
	**(5)** … memory problems
	**(6)** to improve cognitive skills
Other purposes	**(G)** in schools and centers for little children
	**(2)** regarding architecture, to see how a room would look, and the app could also have the ability to measure
	**(3)** In my case, and because I enjoy it, it would be very useful to imagine possible decorations in different spaces
	**(4)** to know your level of orientation skills
	**(5)** experimentation of how certain materials would work in certain environments; decoration; directions on maps, orientation, interaction in certain environments (museums, airports, rooms, etc.), education
	**(7)** it could be very useful to decorate homes
	**(9)** I suppose that for many interesting things
	**(10)** A game, to see how the decoration of a room would look …

*Note*: Each patient's identity was coded with a letter of the alphabet and each healthy participant with a number. The participants with codes ‘D’ and ‘8’ did not answer question 2.

## DISCUSSION

In this study, we examined the feasibility of an AR app to assess object‐location deficits in ABI patients. As expected, ABI patients performed worse than the healthy participants in the AR object‐location memory task across all examined measures (time, success, and attempts). Overall, the perception of the app in terms of how enjoyable and easy it was to use or how much physical and mental effort was necessary, was significantly more positive in the control group than in the ABI patients, although both groups generally had a positive perception of the app. This result is consistent with our initial hypothesis. There were also group differences observed when participants were queried about their preferences regarding the app, suggestions for its application in alternative contexts, and the correlations identified among participants' characteristics, task performance, and subjective perceptions. ABI patients highlighted the entertainment aspects of the app and mentioned its therapeutic potential. Additionally, the control group suggested a greater variety of alternative uses for the technology.

### Performance in the AR task: Brain‐injured patients vs healthy participants

Our results showed that patients with difficulties secondary to brain damage can use an app for smartphones based on AR technology that tests memory for object location. However, this group needed more time to accomplish the task and showed more difficulties in remembering the objects' spatial location than the healthy subjects do. We suggest that the ABI patients' difficulties in performing the task with the application reflect their difficulties in other areas of functioning, such as cognition and/or motor skills. For example, the group differences in the time spent on the task might be due directly to the ABI patient's mobility difficulties. In fact, in the ABI group, the level of dependence significantly correlated with both the time spent performing the task and the level of mobility. However, the increase in the time patients spent compared with the healthy participants can also be explained by their higher number of attempts to place the objects in the correct location.

The results reveal that patients located fewer objects correctly and required more attempts than healthy subjects, which is in line with previous studies describing spatial memory deficits in brain‐injured patients in both near and navigational space (Barrett & Muzaffar, 2014; Cimadevilla et al., 2014; Koen et al., 2017; Monacelli et al., 2003; Piccardi et al., 2011; Rose et al., 1999; Weniger et al., 2009). Accordingly, some authors suggest that successful navigation depends on executive skills (Moffat et al., 2007), which are often altered in ABI patients (Barker‐Collo & Feigin, 2006; Cicerone et al., 2000; McDonald et al., 2002). More specifically, object‐location impairments have been previously reported in patients with brain injuries (Kessels et al., 2002). However, the technology and design used in this latter case differed from those used in our study. The authors used a computer program that allowed them to run a memory task for object location (Kessels et al., 1999). The task was similar to paper‐and‐pencil tests because the objects were pictures shown on a computer monitor for some time. However, as paper‐and‐pencil tests may not accurately reflect daily life functions, some authors highlight the need to use tasks representative of everyday situations to assess cognitive impairments (Pugnetti et al., 1998). Consequently, our AR app obliged users to solve a problem they may find in their everyday routine, namely, remembering the location of common objects in a room. The results of this study suggest that applications based on AR will be sensitive to revealing impairments in spatial memory in populations aged 43–62 years with a brain injury. The results can help design new developments with AR technology for patients with a disability.

### Quantification of user perceptions: Parameters of usability and acceptance of the AR app

Analysis of the participants' subjective factors concerning the AR app revealed that scores obtained by ABI patients were positive but to a significantly lesser extent than those of healthy participants. ABI patients reported significantly more physical and mental effort during the use of the AR app than the healthy group. This result might be due to their limited mobility and deficits in executive functions such as reasoning, planning, mental flexibility, and attention, which have been described previously in ABI patients (Barker‐Collo & Feigin, 2006; Cicerone et al., 2000; McDonald et al., 2002), even when the MMSE scores were higher than 24 (Carelli et al., 2011).

We suggest that deficits in ABI patients' attention could explain not only their higher scores in mental effort, but also their lower concentration during the task. In fact, the correlation analysis in the ABI group revealed a significant relationship between concentration and calm and mental effort, suggesting that the ABI patients' low concentration and high mental effort might be related to the fact that they perceived themselves as less calm during the task. Also, correlation analyses revealed that ABI patients might enjoy the task less because they were less calm and concentrated than the control group.

The results obtained in the UAS show that the ABI group exhibited a positive valuation of satisfaction, concentration, competence, enjoyment, and the usability of the AR app. Their scores were well above the average (5.6, 5.8, 5.4, 5.8, and 5.6 out of 7, respectively). In addition, social influence is also related to the acceptance of technologies (Venkatesh et al., 2003). In our study, the qualitative assessment showed that patients considered that the app contributed socially (e.g., therapy and educational fields). The correlations indicated that satisfaction was positively related to concentration, but there were no direct associations between satisfaction and usability.

Significant differences in usability were observed between groups in favor of the healthy participants. In this regard, a previous study found that for ABI patients, a primary barrier is to learn or to remember how to use a smartphone as assistant technology (Wong et al., 2017) even though, as in our study, smartphone use was equally common in the ABI and the control groups. However, this usage difficulty appears minimized when patients receive direct instructions on how to use the smartphone (Evald, 2015; Wong et al., 2017). The ABI group may have a subjective (but not objective) perception of their difficulties with technology use. In fact, a relationship between high levels of usability and competence, and expertise and low levels of physical effort was found in the ABI group. These correlations suggest that the ABI group perceived the AR app as less usable because they assessed their competence in using technology negatively due to their neurological impairment. Concerning previous studies, low self‐efficacy and anxiety have been observed in older healthy participants with normal cognitive decline when they had to use technological devices for the first time (Czaja et al., 2006). However, no differences between groups were found in how much expertise they felt handling the app. This is a very positive result, suggesting a training effect on the handling of the AR app, regardless of the user's characteristics. It also suggests that potential ABI users would improve their handling through a single instruction session with the AR app.

We found statistically significant differences between the two groups in the level of presence, enjoyment, and satisfaction experienced by the users, in favor of the healthy group. Based on previous research performed with the AR app, we argue that an easy‐to‐learn and easy‐to‐handle app has a positive influence on the level of presence (Munoz‐Montoya, Juan, et al., 2019). In addition, how interesting users find the app, together with their enjoyment when using it, are two factors that also contribute positively to its level of presence (Munoz‐Montoya, Juan, et al., 2019). Supporting previous research (Salar et al., 2020), presence was positively related to the sensory factors of the AR app in the group of patients, but not to other factors related to a positive experience with the app.

The group differences in task performance can explain why some aspects of the AR app were considered less positive by the ABI group compared to the healthy group. As the patients performed worse in the AR task, they were informed about more errors, so they were warned that they should change the way they were thinking during the experience with the app. This fact could explain the increase in their perceived effort and could reduce their calm, concentration, enjoyment, competence, usability, and satisfaction.

In the healthy group, the correlations showed that the less mental and physical effort the participants made, the more they enjoyed the task and the sensory aspects of the app. Considering that healthy participants did not report high levels of mental and physical effort, this might explain why they enjoyed the task more than the ABI group and scored sensory aspects of the AR app higher.

### Qualitative perceptions: Positive aspects of the AR app and uses of AR technology

At the end of the session, participants were asked about the positive aspects of the AR app and ideas they might offer for using the app in other contexts. Regarding what participants liked the most, no differences were observed between groups except for a marginal difference in the entertainment category, in favor of the ABI group. In fact, seven ABI patients commented that the AR app was like a game and that it was fun and enjoyable to use. Considering that enjoyment is a key aspect in accepting an app (Meza‐Kubo & Morán, 2013), AR seems to have the potential to be adopted by ABI patients, similar to what has been described in older adults (Peleg‐Adler et al., 2018).

Concerning possible uses for the app's technology, participants' responses showed group differences. ABI group participants mostly reported the uses of the app for therapeutic purposes. At a cognitive level, their responses may be associated with a reduction in their levels of creativity, which declines after brain damage (Zaidel, 2014). In contrast, the healthy group mentioned a greater number of additional alternative uses.

### Limitations

Three main constraints have to be considered. First, this study uses a small patient sample, which is not representative of all people with ABI. However, recruiting patients with this condition is difficult due to their low incidence rate, the lower cognitive ability of some of the diagnosed cases, and the potential challenges associated with the use of new technologies (Kang et al., 2008; Raspelli et al., 2012; Wald et al., 2000). Second, the limitations presented by the ABI sample determined the degree of difficulty of the task, which was very low for the healthy participants; this limited the number of exploratory correlations. Third, several UAS factors were analyzed on an item level, which limits the reliability and validity of the measurement.

## CONCLUSION

The current results show that the AR app is a promising tool to assess object‐location deficits in ABI patients in tasks that reflect real‐life situations. ABI patients obtained positive scores in usability and acceptance of the app and reported the positive entertainment aspect more frequently than the healthy participants. Future research could examine whether the AR app is an adequate tool for rehabilitation.

## CONFLICT OF INTEREST STATEMENT

The authors declare there are no conflicts of interest.
